# Investigation of the unidirectional spin heat conveyer effect in a 200 nm thin Yttrium Iron Garnet film

**DOI:** 10.1038/srep28233

**Published:** 2016-06-17

**Authors:** Olga Wid, Jan Bauer, Alexander Müller, Otwin Breitenstein, Stuart S. P. Parkin, Georg Schmidt

**Affiliations:** 1 Institut für Physik, Martin-Luther-Universität Halle-Wittenberg, Halle, 06120, Germany; 2 Max Planck Institute of Microstructure Physics, Halle, 06120, Germany; 3 IZM, Martin-Luther-Universität Halle-Wittenberg, Halle, 06120, Germany

## Abstract

We have investigated the unidirectional spin wave heat conveyer effect in sub-micron thick yttrium iron garnet (YIG) films using lock-in thermography (LIT). Although the effect is small in thin layers this technique allows us to observe asymmetric heat transport by magnons which leads to asymmetric temperature profiles differing by several mK on both sides of the exciting antenna, respectively. Comparison of Damon-Eshbach and backward volume modes shows that the unidirectional heat flow is indeed due to non-reciprocal spin-waves. Because of the finite linewidth, small asymmetries can still be observed when only the uniform mode of ferromagnetic resonance is excited. The latter is of extreme importance for example when measuring the inverse spin-Hall effect because the temperature differences can result in thermovoltages at the contacts. Because of the non-reciprocity these thermovoltages reverse their sign with a reversal of the magnetic field which is typically deemed the signature of the inverse spin-Hall voltage.

In 2013 An *et al*. have shown that by the excitation of nonreciprocal spin waves, so-called Damon-Eshbach modes (DEM), in a 400 *μ*m thick Yttrium Iron Garnet (YIG) crystal heat can be transported independent from existing temperature gradient^1^. The direction of the heat flow can be reversed by reversing the applied magnetic field. In their experiments they observed two different effects, both based on the non-reciprocity of the DEM, however, with different consequences. On one hand the asymmetric excitation and propagation of the spin-waves leads to an asymmetric temperature profile which is dominated by the energy loss of the spin waves. These losses are highest at the point of excitation and decrease with increasing distance from this point because the amplitude of the spin-waves decreases. Nonetheless, the energy transport still occurs from the point of higher temperature towards lower temperatures, however, with a certain asymmetry with respect to the source because of the asymmetric propagation of the spin-waves. In an infinite sample no further effects would be observed. If on the other hand the sample is small enough for the spin-waves to actually reach the edge of the sample an additional effect occurs. In this case the spin waves, due to the non-reciprocity cannot be reflected and thus deposit all remaining energy as heat at the boundary. This results in an increase in temperature realizing actually heat transfer by magnons into a warmer region and thus along the temperature gradient as stated by An *et al*. (normally heat transport is against the temperature gradient). In 2015 more detailed theoretical descriptions have been published based on a phenomenological theory^2^ and on micromagnetic simulations^3^. The measurements reported so far were performed with an infrared camera on YIG films with thicknesses ranging from a few micrometers to hundreds of micrometers.

Current research in magnonics concentrates more and more on thin ferromagnetic films and often uses the measurement of the inverse spin-Hall-effect which relies on the measurement of very small voltages. It is thus quite important to know whether a similar scenario can also occur in ultra thin films ultimately leading to temperature gradients which can lead to thermovoltages whose sign might then also depend on the magnetic field.

The DEMs used in the experiment are nonreciprocal surface spin waves^4^. They propagate in opposite directions (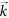 and 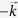) at the top and bottom surface of the layer, respectively^1^,^2^. The precessional amplitude of the DEM is maximum at the surface and decays exponentially inside the film^5^. In order to cause unidirectional heat flow the population of 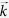 and 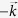 must be different. In a thick sample this condition is realized by exciting the spin waves using a microwave antenna^1^ which is in contact with one side of the layer. In this case the spin waves at the surface close to the antenna are excited more strongly then at the other side resulting in a net spin wave current in one direction.

For thin films with a thickness of a few hundred nm the situation is quite different. The distribution of the precessional amplitude across the film thickness is almost uniform^5^ as well as the excitation by the antenna at the top and bottom surface described above. Nevertheless, the population and propagation of the 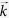 and 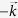 vectors can still be different. The bottom surface of a thin YIG film which is typically grown on gadolinium gallium garnet (GGG) is in contact with the paramagnetic substrate while the top surface is in contact to air. Due to this fact the damping of the spin waves at the top and bottom surface can be different, leading to increased damping of the spin waves propagating in one direction. Also the excitation by the antenna can be nonuniform for thin films. If a waveguide is used for excitation in the Damon-Eshbach geometry the in-plane and the out-of-plane component of the microwave magnetic field can both excite spin waves. The interference of these waves is destructive for k-vectors in one direction perpendicular to the antenna and constructive for the opposite direction^5^,^6^. While in thick YIG layers the spin waves can propagate over millimeters or more^1^,^5^, the spin wave decay length of thin YIG films is typically in the sub-millimeter range^7^ due to enhanced damping for example caused by surface or interface imperfections^8^. Due to this fact we do not expect to observe the heat pile up at the sample edges that An *et al*. describe.

Thus in thin YIG films the unidirectional spin wave heat conveyer effect is expected to be present in terms of an asymmetric temperature profile, however, it will be small in magnitude. Steady-state infrared cameras used in the measurements reported so far are most likely not sensitive enough to detect the effect in thin films. It is, however, possible to use lock-in thermography (LIT), which is well established for failure analysis in integrated circuits^9^ and the characterization of solar cells^10^,^11^. The LIT is a dynamic method, which detects temperature modulation in infrared images similar to electrical measurements using a lock-in amplifier. With this technique the difference in temperature between an excited and a non excited state is imaged. Temperature differences as small as 100 *μ*K can be resolved which is sensitive enough for the small effects described above as we will show later. Details about the used LIT system can be found in the Methods Section.

## Experimental Setup

**Figure 1:**
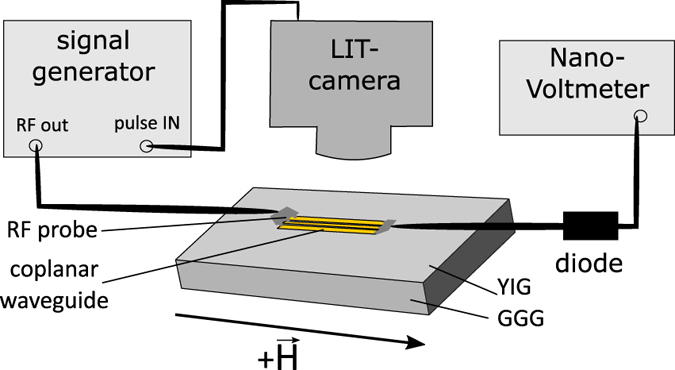
Experimental setup for the FMR and the LIT measurements. The FMR measurements are performed by applying a continuous microwave with a constant frequency and sweeping the magnetic field. For the LIT measurements the microwave has to be pulsed with the lock-in frequency, which is provided by the camera.

A sketch of our experimental setup is shown in Fig. 1. We use a 200 nm high quality YIG film on Gadolinium Gallium Garnet (GGG) substrate grown by liquid phase epitaxy. The damping in ferromagnetic resonance (FMR) *α* of the layer is smaller than 1 × 10^−4^. To excite spin waves we use a coplanar waveguide (CPW) as an antenna, which is fabricated on top of the sample (size of the sample: 5 mm × 8 mm). Details about the fabrication and dimensions of the CPW can be found in the Methods Section.

In order to investigate the spin-wave spectrum FMR measurements are performed. The corresponding experimental setup is shown in Fig. 1. Measurements are done by applying a continuous microwave to the antenna and measuring the transmitted signal using a diode and a nanovoltmeter while sweeping the magnetic field.

For the LIT measurement the camera is placed above the sample and the lock-in reference frequency provided by the camera is used to pulse the microwave excitation.

## Ferromagnetic resonance (FMR) measurements

**Figure 2:**
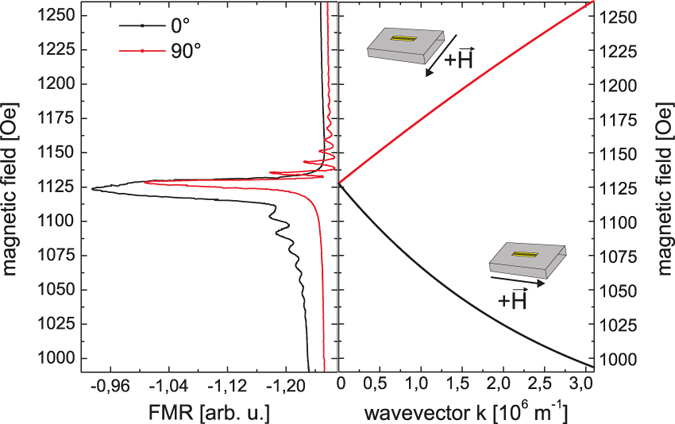
Result of the FMR measurement at 5 GHZ and the calculated dispersion relation for a 200 nm thin YIG film using the following values: saturation magnetization 4*πM*_0_ = 1700 Oe, gyromagnetic ratio *γ* = 2.8 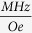.

FMR is measured at a constant frequency of 5 GHz and an excitation power of −1 dBm while sweeping the external magnetic field from 990 Oe to 1260 Oe. The external field is always aligned in the plane of the layer and is either kept parallel to the antenna (0°) to excite the Damon-Eshbach mode with 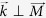 (DEM-geometry) or perpendicular to the antenna (90°) to excite the backward volume mode with 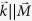 (BVM-geometry). The result of the two respective FMR measurements are shown in Fig. 2 together with the calculated dispersion relation for an in-plane magnetized 200 nm YIG film. These dispersion curves for dipolar spin waves have been calculated using the equations given in^5^. For *k* = 0 the uniform mode is excited at a field of 1128 Oe. For *k* ≠ 0 the dispersion relation exhibits two branches, one for the DEM at lower fields and one for the BVM at higher fields. Comparing the FMR spectra to the dispersion relation we can see that for both geometries the uniform mode is excited. In the DEM geometry we also observe a signal at lower 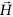 while for the BVM-geometry resonances at higher 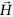 appear in good agreement with the calculation. We do, however, not observe the expected continuous spin-wave spectrum but several resonance lines. These lines appear because the geometry of the waveguide favours certain k-vectors corresponding to a fundamental k-value and integer multiples with decreasing amplitude. It should be noted that the DEM with the highest intensity overlaps with the uniform mode and is not visible as a separate peak.

## Lock-in thermography measurements

### Damon-Eshbach geometry

**Figure 3:**
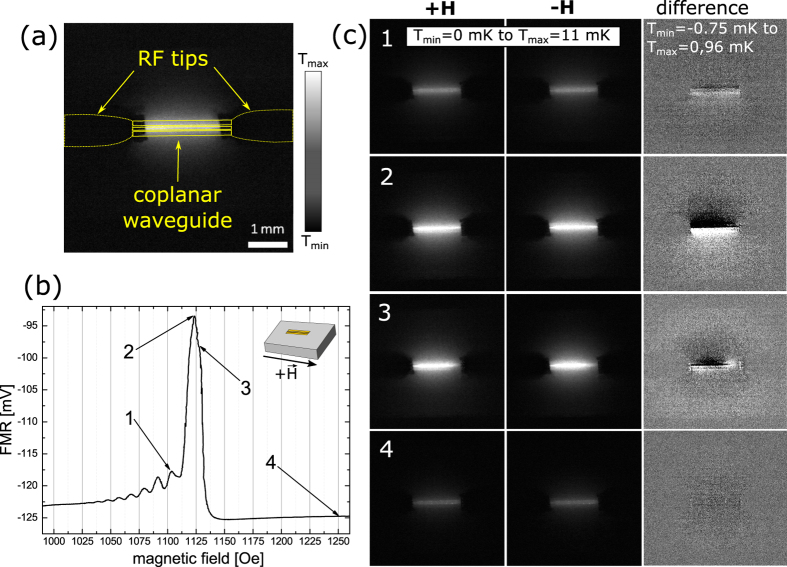
(**a**) LIT amplitude image with a sketch of the position of the CPW and the rf tips. (**b**) FMR measurement in the Damon-Eshbach geometry (**c**) LIT measurements when the magnetic fields 1, 2, 3, 4 and the corresponding negative values are applied (left and center). Black corresponds to no increase in temperature while white indicates an increase of 11 *mK*. The calculated difference (right) shows the expected effect. It should be noted that in the difference images the gray scale has been changed for better visibility. The range is now between −0.75 mK (black) or lower and 0.96 mK (white) or higher.

For the lock-in thermography measurements we use the knowledge obtained from the ferromagnetic resonance spectra. First the DEM-geometry is investigated where the external H-field is applied parallel to the antenna (0°). Using different field values we excite spin waves of different k-vectors and take images with the camera. Figure 3(a) shows an amplitude image taken by the LIT camera at a magnetic field of approx. 1120 Oe including a sketch of the rf tips connected to the coplanar waveguide. For the interpretation of the image it is important to first understand what is actually visible. As a typical lock-in technique the LIT is able to eliminate background signals. In this case the absolute temperature is not measured. The grey scale of the images is thus a scale for the temperature difference between the state without and with excitation (or in other words the temperature increase by the excitation) and is measured with high accuracy. We do not expect to measure negative values because no active cooling is expected in our case. The background which is eliminated by the lock-in technique corresponds to room temperature. The accuracy of the measurements has also been determined by comparing different measurements and we can show that the error is typically 0.15 *mK* or less. Error bars are thus omitted when temperature profiles are displayed in graphs. It should be noted that the local emissivity can lead to deviations of ±5 − ± 10% which can locally distort the temperature profile. However, as we will see later, we obtain our results mainly from the difference between two measurements in which the systematic error of the emissivity is eliminated.

In our experiment the magnons traveling away from the waveguide lose their energy to the lattice^1^ and the LIT camera measures the resulting change in the lattice temperature. As the magnons are damped along the direction of propagation the magnitude of the transfer is smaller further away from the waveguide leading to a decay of the temperature difference with the magnon intensity. These measurements are repeated at different magnetic fields which are marked in Fig. 3(b). The values shown correspond to (1) the DEM peak with second largest intensity and no overlap with the uniform mode, (2) the maximum DEM peak overlapping with the uniform mode, (3) the uniform mode itself, and (4) a position completely off resonance where no excitation is expected at all, respectively. All measurements are repeated after reversing the direction of the magnetic field. It should be noted, that when the magnetic field is reversed the FMR spectrum remains the same as a function of the absolute value of the field.

**Figure 4:**
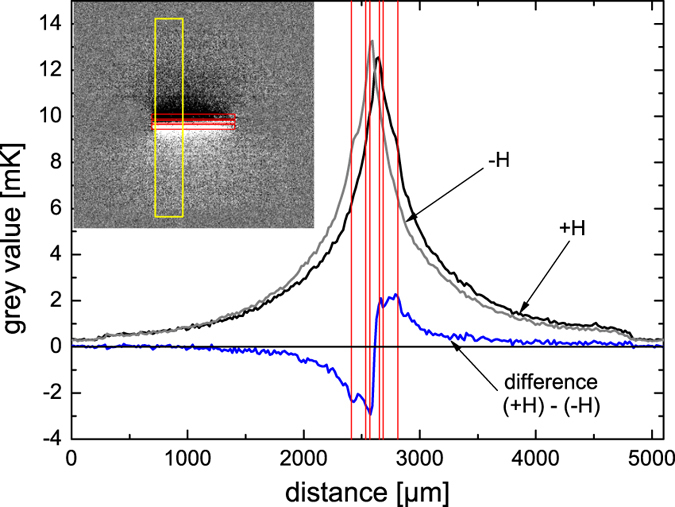
Temperature profile plotted for the yellow marked region. Red lines show the position of the CPW.

Figure 3(c, column +H and −H), show the images taken for opposite H-field directions for the respective magnetic fields. Already in these pictures we can clearly see that the heating scales with the absorption amplitude. The corresponding values for the measured RF absorption and the respective increase in temperature are: 7 mV/5.2 mK (pos. 1), 31.2 mV/12 mK (pos. 2), 26.1 mV/11.6 mK (pos. 3), and 0 mV/3.4 mK (pos. 4). The absorption was determined by subtracting the off resonance voltage at high magnetic fields from the voltage measured at the respective magnetic field. The small heating off-resonance stems from the ohmic losses in the antenna. In order to extract the spin heat conveyer effect from the images, however, a special procedure is necessary. The non-reciprocity of the DEM results in enhanced spin wave emission and propagation on one side of the antenna. This preferential propagation should lead to heating profile which is asymmetric in the direction perpendicular to the antenna. The asymmetry, however, is very small with respect to the total heating and can not or only barely be observed on the grey scale of the unprocessed LIT images. In order to visualize the effect we make use of the fact that the preferential propagation direction and thus the asymmetry should be reversed with the reversal of the magnetic field while, as will be discussed below, any artifacts and the heating background do not depend on the field direction. We can thus subtract the images for positive and negative fields and obtain as a result images which only show the profile of the asymmetry (Fig. 3(c), difference images in right column). Obviously in these pictures we can also see negative values. These do not indicate cooling but just occur when the local temperature in the subtracted image is larger than the one from which is subtracted. In fact the difference profile should be completely antisymmetric because we subtract two asymmetric profiles which are expected to be mirror inverted with respect to the antenna. Figure 4 illustrates the procedure. It shows temperature line profiles obtained by line-wise averaging the data inside the yellow region shown in the inserted image for positive (black curve) and negative (grey curve) H-field, respectively. For the diagram the grey value is converted to the temperature in mK. Already here we can observe that temperature profile close to the antenna is higher on one side than on the other resulting in a temperature profile which in total is asymmetric. This asymmetry is reversed when the magnetic field changes sign. When we plot the difference between the two graphs (blue curve) we find an antisymmetric profile as expected. This profile shows that the maximum temperature difference can be as big as 2.6 mK and decays to zero far away from the antenna as expected from theory. It should be emphasized that the fact that we can observe the asymmetrical temperature distribution also outside of the region of the antenna (red lines in Fig. 4) clearly shows that the effect indeed originates from propagating spin waves.

If we now analyse the difference images in detail we find the following. For the first DEM (smaller amplitude) only a small asymmetry can be observed (position 1, Fig. 3(b,c)). Moving to the maximum intensity of the DEM (position 2, Fig. 3(b,c)) not only yields a much bigger increase in temperature but here also the difference image clearly shows an antisymmetric temperature profile extending over several mm across and beyond the antenna. For the uniform mode at line position 3 (Fig. 3(b,c), 3) the heating should be symmetric and independent from the direction of the magnetic field. Surprisingly, we still observe a small asymmetry which results from the overlap with the DEM at position 2. This effect will later be discussed because it is highly relevant for measurements of the inverse spin-Hall effect (ISHE)^12^. As expected we only observe little heating and a homogeneous temperature distribution for the off-resonance measurement (Fig. 3(b,c), 4).

The evaluation procedure described above also helps to eliminate possible side effects and artifacts: first the influence of the antenna should be discussed. As also outlined in the Methods Section the whole sample surface is covered by black ink to ensure uniform emission properties for heat radiation eliminating for example the low radiation efficiency of a blank metal surface. The question remains, however, whether in the areas of metallization the images really show the temperature of the YIG or of the metal and whether any heating really stems from the resonance in the oxide layer rather than from losses in the coplanar waveguide. The latter can easily be excluded by comparing pictures off-resonance and on-resonance which indicate that the heating by the antenna observed off-resonance is much smaller than the heating by the resonance in the YIG. In addition, this heating does not change during a field reversal and can thus be eliminated from the measurements by using the difference image between opposite magnetic fields. Visibility of the YIG temperature through the metal is also guaranteed because the thickness of the metal is only a few hundred nm and the materials of the coplanar waveguide (Ag and Au) have very high heat conductivity. At the modulation frequency of 1 Hz the top of the antenna can always be considered at the same temperature as the YIG surface underneath. Lateral heat conduction in the thin metal film, however is much lower and will only slightly smear out any temperature profile originating from the spin waves. The same holds for heat diffusion in the YIG. Anyway, both, heat diffusion in metal and YIG are again independent from the magnetic field and can thus be eliminated by using the difference of two images with opposite field directions. The smearing can, however, slightly reduce the effect that we intend to observe.

### Backward Volume geometry

**Figure 5:**
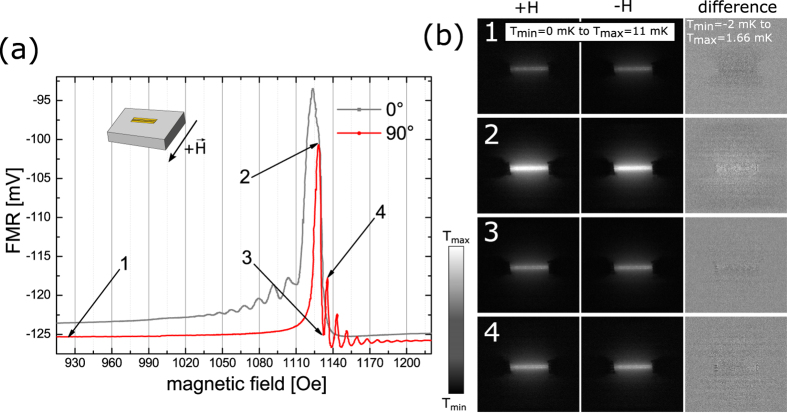
(**a**) FMR measurement in the Backward Volume geometry (90°, red curve) compared to the FMR measurement in the Damon-Eshbach geometry (0°, grey curve). The peaks of the BVM appear much sharper than for the DEM. (**b**) LIT images for the magnetic field positions 1 to 4.

In a next step the BVM geometry (90°) is investigated where the external magnetic field rotated by 90° with respect to the DEM geometry. The field is still in the plane but now perpendicular to the antenna. In the BVM geometry a similar set of measurements is done as for DEM, now sampling the field range of the backward volume modes again including the uniform resonance mode. In the Backward Volume geometry no non-reciprocal spin-waves can be excited, so that no unidirectional heat transfer should be observed. Figure 5 displays the spin wave spectrum together with the LIT images again for four different field values. For all cases the unprocessed LIT images show heating similar to the respective measurements in DEM geometry scaling with the RF absorption: 0.3 mV/4 mK (pos. 1), 24.8 mV/11 mK (pos. 2), 0.6 mV/5.3 mK (pos. 3), and 7.8 mV/6.5 mK (pos. 4). The difference images, however, either show only a very small difference in temperature or none at all and lack the asymmetry observed for the DEM. The finite difference appearing especially for pos. 2 does not originate from non-reciprocity of the spin waves but from a minute difference in heating for opposite fields. As the FMR data shows the resonance lines in the BVM-geometry are very sharp. When the magnetic field is reversed there is always a small but finite difference in the absolute absorption which stems from the finite offset of the field measurement and the finite resolution of the field controller. Even a difference of 0.1 Oe in the field (corresponding to a relative error of 100 *ppm*) which is barely detectable in the FMR measurement can lead to a small difference in heating. This does not lead to a different shape of the heating profile but only to an overall increase or decrease by a scaling factor. As a result we observe a small temperature difference in the difference image. The maximum temperature difference observed here is as small as 0.2 mK compared to a total heating of 11 mK and is only visible because of the high sensitivity of LIT. Inhomogeneities in the profile (corresponding to less than 0.1 mK) result from noise and small local inhomogeneities of the heat emission. It should again be noted that here the two difference images show either a positive difference or a negative difference while in the case of the DEM the difference is always antisymmetric showing both positive and negative values.

## Discussion

Our results show that LIT is well suited to assess the local heating of our samples even on the mK scale and below. We clearly observe that as soon as spin wave modes are excited no matter whether in the DEM or in the BVM geometry the magnons heat the YIG causing a heating profile that decays away from the waveguide corresponding to the damping of the magnons. The heating scales with the RF absorption in the YIG layer. Heating is observed in both the DEM and the BVM geometry and is also of similar magnitude in both cases. However, while the heating itself does not depend on the field geometry the shape of the heating profile does. Only in the DEM geometry do we observe an asymmetry of the profile which can be explained by the non-reciprocity of the DEM. In this respect it is especially interesting to analyse the measurements at the magnetic field value where nominally the uniform mode is excited. At this field the amplitude of the FMR measurement is high in the DEM geometry and maximum in BVM geometry. It is important to note that in both cases a large area around the waveguide is heated showing that besides the uniform mode also propagating spin waves are excited. However, only for the DEM geometry we see an asymmetric heating. For the BVM both sides of the antenna are equally heated and the heating profile is fully symmetric although the value of the resonance field is the same as for the DEM. Our results thus show that even for thin YIG films the unidirectional spin wave heat conveyer effect can be observed when the external field direction satisfies the condition for the excitation of DEM. In the DEM geometry even low amplitude spin waves lead to small temperature asymmetries around the waveguide, while at high amplitudes the temperature asymmetry is significant and extends several mm beyond the boundaries of the antenna. We do, however, not observe the heat pile-up at the sample boundaries shown by An and coworkers because in our thin films the magnons do not reach the edges of the sample. Only at the edges the non-reciprocal spin waves cannot be reflected and thus deposit their energy like a beam of radiation hitting a non-reflecting surface. Although the temperature differences and the lateral extent of the effect are smaller than for thick YIG films, lock-in thermography allows us to clearly identify the effect. The magnitude of the signal that is observed indicates that it should also be present in even thinner layers which nowadays can also be obtained with very low damping (for example^13^,^14^,^15^) and may possibly be detected using longer averaging times. The fact that this effect needs to be considered also in thin films has important consequences for other research areas. The DC-ISHE is typically measured in a DEM geometry. Our experiments now show that in this geometry even the uniform mode can create a temperature gradient on the length scale of several mm along the direction in which the ISHE voltage is measured. If now the voltage probes used to measure the ISHE make contact in places of different respective temperature each contact represents a thermocouple and a thermovoltage can be detected in between. Due to the non-reciprocity of the DEM this thermovoltage changes sign upon reversal of the magnetic field. Up to now this sign reversal is considered as undisputable proof for the ISHE and only the Nernst effect is considered as a possible candidate for magnetic field dependent DC signals^16^. From our measurements we conclude that at least for materials with high Seebeck coefficients or extremely small ISHE voltages it is necessary to either investigate both effects, thermovoltages and ISHE, separately in order to quantify the contribution from the temperature gradient^12^ or to make sure that the voltage probes are place in isothermal spots. In particular when organic materials are used the possibly large Seebeck coefficient might result in voltages as high as those observed for the ISHE. The conducting Polymer poly(3,4-ethylenedioxythiophene) polystyrene sulfonate (PEDOT:PSS) which is often applied in organic electronics can for example exhibit a Seebeck coefficient of 160 *μVK*^−1^ or higher^17^,^18^ resulting in a thermovoltage of 160 nV for a temperature difference of 1 mK. For thick YIG layers where the heat conveyer effect is much more pronounced the expected thermovoltages can thus be much bigger. This effect will be experimentally investigated in the future.

## Methods

### Fabrication of the CPW

The CPW is fabricated on top of the YIG film using electron beam lithography, metal deposition (10 nm Ti/250 nm Ag/50 nm Au) and lift-off. The dimensions of the waveguide are as follows: length = 2.9 mm, width of the signal line = 80 *μ*m, width of the ground planes = 125 *μ*m, distance between signal line and ground planes = 35 *μ*m.

### Ferromagnetic resonance (FMR) measurement

All FMR measurements shown in this paper have been performed using a microwave frequency of 5 GHz and a excitation power of −1 dBm, by sweeping the magnetic field. The external magnetic field is applied by an electromagnet which can be rotated around the sample. The microwave is provided by a RHODE&SCHWARZ, SMF 100 A signal generator, which is connected via rf probes (Cascade Microtech) to the CPW on top of the YIG sample. While sweeping the magnetic field at a constant frequency we measure the absorption using a Schottky diode and an Agilent 34420 A nanovoltmeter.

### Lock-in thermography technique

For the LIT experiments we used an InfraTec PV-LIT system, which works with an InSb detector having a resolution of 640 × 512 pixels (www.infratec-infrared.com; Accessed: 28th April 2016). To perform the LIT measurement the microwave power is pulsed at the lock-in frequency supplied by the camera. For all LIT measurements shown in this paper we use an acquisition time of 5 minutes and a lock-in frequency of 1 Hz. The surface of the sample is blackened with ink to achieve a better and uniform infrared emissivity. In a LIT experiment the heat sources in a device are modulated or pulsed at a lock-in frequency lying well below the frame rate of the infrared camera (here 200 Hz). The acquired images are evaluated synchronously to the heat pulses to detect the temperature modulation. Both the in-phase and the out-of-phase modulations are detected and can be converted into an amplitude and a phase signal. This is done on-line for each pixel. The result is equivalent to connecting each pixel to a two-phase lock-in amplifier^9^,^10^,^11^. To achieve maximum precision when subtracting images taken for positive and negative field values corresponding to sharp peaks in the resonance we perform the following procedure. The magnetic field is first swept over the peak under investigation. Then the same sweep is done again and is stopped when the maximum of the peak is reached. Only then the LIT image is acquired. This way the influence of a small but finite offset of the magnetic field measurement can almost be completely eliminated which otherwise could lead to differences in absolute heating as described above for the BVM.

## Additional Information

**How to cite this article**: Wid, O. *et al*. Investigation of the unidirectional spin heat conveyer effect in a 200 nm thin Yttrium Iron Garnet film. *Sci. Rep*. **6**, 28233; doi: 10.1038/srep28233 (2016).
